# Metal-Based Nanoparticles for Cardiovascular Diseases

**DOI:** 10.3390/ijms25021001

**Published:** 2024-01-13

**Authors:** Alexandru Scafa Udriște, Alexandra Cristina Burdușel, Adelina-Gabriela Niculescu, Marius Rădulescu, Alexandru Mihai Grumezescu

**Affiliations:** 1 Department 4 Cardio-Thoracic Pathology, “Carol Davila” University of Medicine and Pharmacy, 050474 Bucharest, Romania; 2 Department of Science and Engineering of Oxide Materials and Nanomaterials, Politehnica University of Bucharest, 011061 Bucharest, Romaniaadelina.niculescu@upb.ro (A.-G.N.);; 3 Research Institute of the University of Bucharest—ICUB, University of Bucharest, 050657 Bucharest, Romania; 4 Department of Inorganic Chemistry, Physical Chemistry and Electrochemistry, University Politehnica of Bucharest, 1-7 Polizu St., 011061 Bucharest, Romania

**Keywords:** cardiovascular diseases, nanomedicine, metal-based nanoparticles, iron oxide nanoparticles, gold nanoparticles, silver nanoparticles, cerium oxide nanoparticles

## Abstract

Globally, cardiovascular diseases (CVDs) are the leading cause of death and disability. While there are many therapeutic alternatives available for the management of CVDs, the majority of classic therapeutic strategies were found to be ineffective at stopping or significantly/additionally slowing the progression of these diseases, or they had unfavorable side effects. Numerous metal-based nanoparticles (NPs) have been created to overcome these limitations, demonstrating encouraging possibilities in the treatment of CVDs due to advancements in nanotechnology. Metallic nanomaterials, including gold, silver, and iron, come in various shapes, sizes, and geometries. Metallic NPs are generally smaller and have more specialized physical, chemical, and biological properties. Metal-based NPs may come in various forms, such as nanoshells, nanorods, and nanospheres, and they have been studied the most. Massive potential applications for these metal nanomaterial structures include supporting molecular imaging, serving as drug delivery systems, enhancing radiation-based anticancer therapy, supplying photothermal transforming effects for thermal therapy, and being compounds with bactericidal, fungicidal, and antiviral qualities that may be helpful for cardiovascular diseases. In this context, the present paper aims to review the applications of relevant metal and metal oxide nanoparticles in CVDs, creating an up-to-date framework that aids researchers in developing more efficient treatment strategies.

## 1. Introduction

Comprising a series of conditions that affect the heart and blood vessels, cardiovascular diseases (CVDs) rank among the world’s primary causes of death and disability for people [1,2]. According to the World Health Organization (WHO), more than 18.6 million deaths, accounting for 31% of all deaths worldwide in 2019, were attributable to CVDs covering a broad range of disorders, including myocardial infarction, ischemic heart disease, coronary artery disease, congestive heart failure, and stroke. Thus, creating efficient non-invasive imaging techniques for early diagnosis and monitoring the ensuing therapy response of CVD treatment has become crucial to handling this dire situation [3,4].

Numerous innovative nanomaterials and nanodevices for diverse scientific and medicinal applications have been made possible by nanoscience, nanotechnology, and nanomedicine advances [5,6,7]. Nanoparticles (NPs) benefit from several advantageous features: small dimensions, large surface area, and tunable shape and morphology being among the most important [8,9]. NPs can be made from various chemical compositions, as a wide range of possible materials have been fabricated by researchers worldwide. Formulations based on polymeric, liposomal, inorganic, or hybrid nanomaterials have been increasingly investigated in recent years as emerging diagnosis and treatment options for various diseases [4,6,7,9,10,11].

Out of the plethora of potential nanomaterials for biomedical use, cardiovascular medicine has shown particular interest in metal-based NPs because of their distinct electrical and visual characteristics. Smaller ones, ranging in size from 10 to 100 nm, interact well with biomolecules on the surface and inside cells. In addition to possible biocompatible polymers, the surface area can be altered with a range of functional groups to target different cells, including peptides, antibodies, medications and enzymes, RNA, and DNA [4,12]. Metal NPs can potentially be theranostics for treating CVDs due to their high therapeutic efficacy at diseased locations and ability to transport drugs with precision [5,13,14].

Notwithstanding several possible hazards about their long-term safety, metal-based nanoparticles have already established themselves in several biomedical applications. Various cardiovascular problems are assessed, evaluated, and treated with new diagnostic, therapeutic, and prognostic methods [15]. In recent decades, nanomedicine has drawn attention as a crucial, safe, and effective tool that can be used to treat angiogenic, inflammatory, ischemic, and metabolic diseases like hypertension, atherosclerosis, and hyperlipidemia [4,5,16]. Furthermore, drug delivery, wound healing, stem cell applications, imaging methods, and medical tool design are all presently using nanomedicine [17]. This significant advancement expands on the previously identified uses of NPs, which include their antibacterial, antiviral, and antifungal properties [18,19,20]. Similarly, nanotechnologies have made comprehensive DNA analysis and genomic detection possible [4,21].

**Figure 1:**
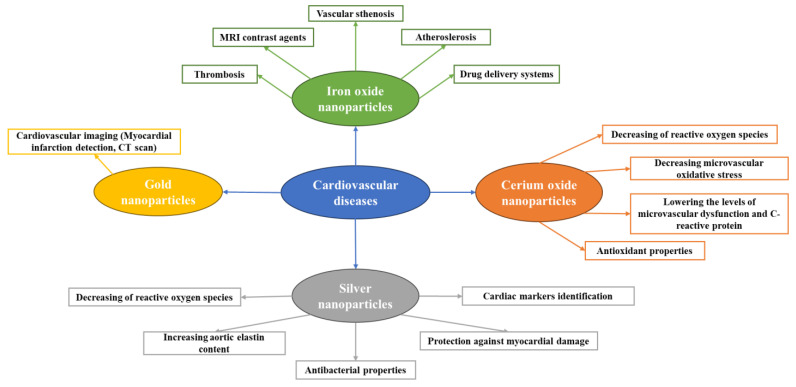
Metal-based nanoparticles usage in CVDs. Created based on information from [4,15,22].

Due to their unique characteristics, metal and metal oxide NPs may have various effects on the control and therapy of CVDs [22]. The FDA has approved genetic technologies based on gold nanoparticles (AuNPs). They are currently inferred from the identification of several molecular and genetic biomarkers. Moreover, other NPs, such as superparamagnetic iron oxide nanoparticles (SPIONs), are considered safe and efficient MRI contrast agents for therapeutic evaluation or targeted molecule targeting. It is interesting to note that careful control over the physicochemical characteristics of NPs significantly speeds up the development of patient-specific medical interventions [23,24]. In contrast to their growing use in cancer [25,26,27] and hereditary diseases [28,29,30], NPs are still relatively new when managing or treating CVDs, yet they have been found promising for a series of applications [4], as depicted in Figure 1.

In this context, this review has gathered information on metal-based NPs, highlighting their cardiovascular effects and promising potential as CVD therapeutics. Because iron oxide, cerium oxide, gold, and silver nanoparticles have been extensively studied for their biomedical properties in animal and in vitro studies [4,31], we further discuss their significance in managing CVDs. We also provide information on the use of these NPs in the production of synthetic cardioprotective molecules, the delivery of medications and stem cells, and the detection of CVD diseases.

## 2. Iron Oxide Nanoparticles (IONPs)

Over the past few decades, magnetic NPs have been widely employed in biomedical applications, such as cell-sorting assays, magnetic resonance imaging (MRI), and immunoassays, being also used as contrast agents [32,33,34,35]. When compared to cobalt (Co) iron (Fe) nanomaterials, iron oxide nanoparticles (IONPs) are used more frequently because of their lower specific loss of power, lower saturation magnetization, and lower magnetic properties [36]. Table 1 presents the most important types of iron nanoparticles relevant to CVD management and their applications, which will be further discussed in this section.

### 2.1. Various Iron Oxide-Based Nanoparticles

It has been demonstrated that using functionalized IONPs with particular targeting capabilities enhances the MRI signals for vulnerable and early-stage atherosclerotic plaques [37]. There is a growing interest in further tailoring implantable image neuroprostheses to facilitate therapeutic functions, including dual diagnosis and therapy. One strategy is to load therapeutic components concurrently with targeting molecules. Certain ligands were used to target early or advanced plaques, while others were combined to increase targeting efficacy [38]. Furthermore, magnetic NPs may be used for other CVD diagnosis techniques, such as near-infrared fluorescence and ultrasound, improving visualization beyond MRI [37].

In addition, IONP-based formulations can serve as novel drug delivery systems for better managing CVDs. For instance, phase transition or biomimicking could be applied in place of standard drug delivery strategies for enhanced treatment outcomes. In this respect, Banik et al. [39] synthesized dual-targeted, lipoprotein-mimicking synthetic therapeutic IONPs with surface functionalities for targeting macrophage mannose receptors and mitochondria. They showed in vitro and in vivo lipid removal and MRI contrast-enhancement capabilities.

Differently, Fe_3_O_4_-PFH-DiR@CS-DS NPs that react to low-intensity focused ultrasound (LIFU) were engineered by Hou et al. [40], who have rationally designed and engineered the phase transition agent perfluorohexane (PFH)-responsive FPD@CD nanomedicine for the highly effective treatment of vulnerable plaques. This is achieved by easily loading PFH into biocompatible PLGA-PEG-PLGA nanoparticles (PPP NPs), which are then surface-attached with dextran sulfate (DS) for targeted delivery. Since DS is a typical macrophage-targeted molecule, it can precisely vaporize NPs, which in turn causes RAW 264.7 macrophages to undergo controllable apoptosis due to the acoustic droplet vaporization (ADV) effect. Furthermore, the advent of DiR and Fe_3_O_4_ gives nanomedicine the ability to perform magnetic resonance (MR) imaging and near-infrared fluorescence (NIRF). Based on results from in vitro and in vivo evaluations, the engineered FPD@CD nanomedicine, which targets macrophages as therapeutic targets, has the obvious therapeutic effect of reducing susceptible plaques. Throughout the treatment (20 days), a 49.4% decrease in the degree of vascular stenosis in gross pathology specimens was attained. This particular, effective, and biosafe treatment modality enhances the biological application based on the reduction in plaque rupture issues in patients with cerebrovascular and cardiovascular disorders [40]. MRI and near-infrared fluorescence were added to the NPs by incorporating DiR and Fe_3_O_4_. Meanwhile, they could precisely vaporize and control the apoptosis of RAW 264.7 macrophages by coating with macrophage-targeting dextran sulfate and loading with the phase transition agent perfluorohexane [40].

Furthermore, IONPs have been reported as cardioprotective materials. Specifically, Xiong and colleagues [41] have designed and evaluated 2, 3-dimercaptosuccinic acid-modified Fe_2_O_3_ NPs to establish their potential for treating CVDs. The researchers concluded that these nanomaterials represent promising candidates for protecting the heart from ischemic damage at the animal, tissue, and cell level.

There is a constant flow of new techniques. In the framework of recent studies, atherosclerotic plaque was treated and visualized using iron NPs-based chemo- and photodynamic therapies, which are commonly used in tumor medicine [32]. As atherosclerosis, thrombosis, and myocardial infarction (MI) progress, macrophages infiltrate and accumulate, which makes them a viable target for non-invasive methods [42]. By using dextran to modify the surface of IONPs, one can frequently target macrophages by taking advantage of the expression of C-type lectins that bind dextran on the surface of macrophages [43].

Sosnovik et al. [44] demonstrated that by using magneto fluorescent nanoparticles (cross-linked iron oxide (CLIO)-Cy5.5) composed of dextran-CLIO and Cy5.5, macrophage imaging was achievable in mice. Twelve mice were given a left coronary artery ligation to cause MI, and seven mice received a sham operation as a control. Following surgery, CLIO-Cy5.5 was injected, and 48 h later, MRI and fluorescence tomography were carried out. The contrast-to-noise ratio increased (23.0 ± 2.7) in MR images of the infarcted mice but not in those of the sham-operated mice (5.43 ± 2.4). The infarcted mice had a higher fluorescence intensity (19.1 ± 5.2) compared to the control group (5.3 ± 1.4) [44].

A new class of atheroprotective Fe@CNPs that resembled high-density lipoprotein was created by Akhmedov et al. [45]. Gas-phase synthesis was used to create these NPs, which were subsequently modified with lipophilic organic functional groups. When immobilized in vitro on stainless steel using polycaprolactone, Fe@C NPs demonstrated excellent cytocompatibility. Additionally, they could enter the internal structures of atherosclerotic plaques and alter their structural makeup in vivo [45].

On a different note, IONPs were employed as less harmful catalysts for starch oxidation in a recent study published by Hataminia et al. [46]. The thermoplastics made from the modified starch were then used to coat cardiac stents. The resulting nanocomposite showed longer drug release and antioxidant activity even without drugs, and it was more stable than conventional thermoplastics [46].

The iron oxide, which is more commonly known as magnetite (Fe_3_O_4_) or maghemite (γ-Fe_2_O_3_), forms the core of these nanoparticles, with a radius varying from 5 to 15 nm. Since iron oxide is naturally hydrophobic, the nanoparticles are typically altered by adding an external coating made of a biocompatible hydrophilic polymer, like polyethylene glycol or dextran [47]. This coating is necessary to stop the nanoparticles from opsonizing or binding with plasma proteins, which would otherwise inevitably happen in the bloodstream [48]. Furthermore, because of the hydrophobic qualities of the coating, it prevents the aggregation of nanoparticles through electrostatic interactions. It has been proposed that a common underlying mechanism known as myocardial inflammation underlies the development and progression of several cardiovascular diseases [49].

### 2.2. Superparamagnetic Iron Oxide Nanoparticles (SPOIN) and Ultrasmall Superparamagnetic Particles of Iron Oxide (USPIO)

**Figure 2:**
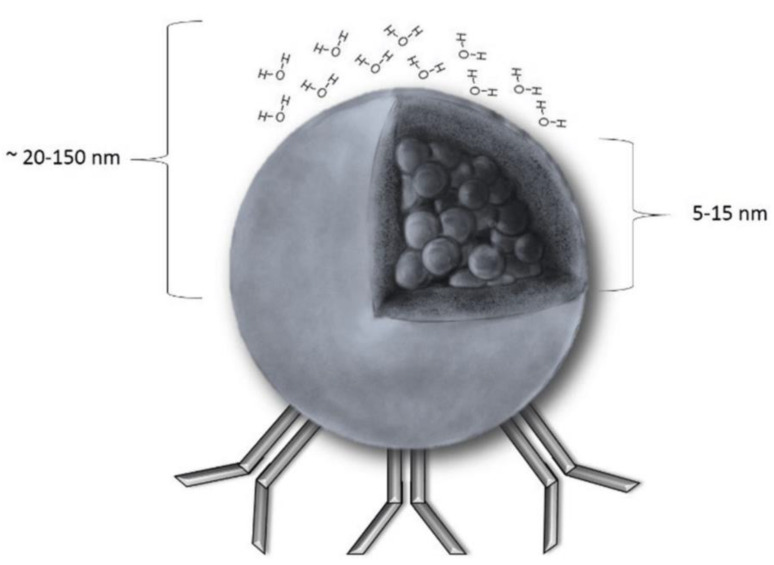
An illustration of a superparamagnetic iron oxide nanoparticle, or SPION: The hydrodynamic radius (core with shell and water coat) is between 20 and 150 nm, while the core radius is between 5 and 15 nm. Magnetization is equal to zero when no magnetic field is present. As demonstrated, most SPION applications found to date are made possible by the ease with which SPIONs can be coupled with antibodies. Reprinted from an open-access source [52].

Superparamagnetic iron oxide nanoparticles (Figure 2) can be chemically synthesized using various techniques or produced as emissions from industry and power plants [32]. The synthesis possibilities include the polyol method, reactions in restricted settings, flow injection synthesis, sonolysis, and classical synthesis by co-precipitation [50]. Furthermore, new techniques for synthesizing nanoparticles have been reported, including producing iron oxide nanoparticles by rapid inductive heating and magnetic α-Fe_2_O_3_/Fe_3_O_4_ heterogeneous nanoparticles through a simple solution combustion process. Two of their most important properties are size and coating, which largely dictate their potential applications, safety, and bioavailability [51].

Cardiac MRI has emerged as a potent, non-invasive modality that permits direct visualization of the heart. Research interest is growing in the emerging application of ultrasmall superparamagnetic particles of iron oxide (USPIOs) and their magnetic properties [49,53]. Since it can detect active inflammation in the myocardium, which has been postulated to be the basis for adverse remodeling and heart failure, USPIO-enhanced cardiac MRI can yield important information.

In Europe and the U.S., ferumoxytol (Feraheme), a USPIO coated with carboxymethyl dextran, is authorized to treat iron deficiency anemia. In addition to treating anemia, ferumoxytol is becoming more popular as an MRI contrast agent, being frequently used in research using approved procedures [54,55,56]. In a clinical trial involving 14 patients with acute MI, Yilmaz et al. [57] assessed whether ferumoxytol allows a better MRI characterization of infarct pathology than gadolinium-based agents. After the onset of cardiac symptoms, baseline cardiac magnetic resonance imaging was carried out starting from 48 h to 7 days later. The patients from the mentioned study suffered from acute ST-segment elevation myocardial infarction (STEMI) due to ischemic myocardial injury and coronary artery occlusion or plaque rupture. Twenty-four hours after the baseline cardiac magnetic resonance study, ferumoxytol (17 mL ferumoxytol containing 510 mg Fe) was injected intravenously. Then, 48 h after ferumoxytol was administered, patients had postferumoxytol studies. According to analyses, ferumoxytol provided a more thorough examination of MI, primarily through macrophage infiltration, and as a result, it may offer a more useful profile for assessing MI than Gd-based agents [57].

In patients at risk of developing nephrogenic systemic fibrosis due to chronic renal disease, USPIO can also be implemented as a safe substitute for gadolinium-based contrast agents. With 79 reported cases of anaphylaxis from an estimated 1.2 million injections, the U.S. Food and Drug Administration issued a boxed warning due to one of the main safety concerns: the possibility of an acute hypersensitivity allergic reaction. Large-scale research has, however, successfully backed up their safe use in clinical practice ever since. Over 3000 patients received over 4000 ferumoxytol injections in a large multicenter MRI registry; only 0.2% of patients experienced moderate adverse events [58]. No severe or fatal adverse events were reported. The safety profile of USPIO has improved in the past few years, and evidence from extensive studies continues to support their safe and appropriate use in clinical practice. Significant mechanistic pathways involving cellular inflammation and active macrophage infiltration have been successfully identified through their application in CMR. The research findings thus far demonstrate their successful application in the study of cardiac diseases and the potential that USPIO-enhanced CMR holds for application in clinical practice, direct treatment approaches, and central to personalized medicine [52].

In addition, IONPs are one nanomaterial that has shown great promise in supporting stem cell therapy among other forms of cell therapy. Through functionalization, they provide non-invasive tracking and monitoring of transplanted cells and disease progression and improve stem cell transplantation, differentiation, and proliferation. The enormous potential of stem cells and their exosomes in myocardial repair, including the treatment of MI and cardiomyopathy, has been demonstrated by earlier experimental and preclinical research [59]. On the other hand, their limited ability to recruit cells to the ischemic area naturally may limit their efficacy in myocardial repair. An increasing amount of research is being conducted on using IONPs, which can enhance the delivery of stem cells for effective repair while simultaneously imaging them [60]. By loading neutrophils with SPIONs conjugated to an antibody against CD34, a surface marker of endothelial progenitor cells (EPC), SPION-antiCD34/Nes, was created in a recent study by Sun et al. [61]. By releasing preloaded EPC-binding molecules, this method effectively captured EPCs at lesions by utilizing neutrophils’ targeting ability. In vivo, SPION-antiCD34/Nes demonstrated acceptable biocompatibility. In a mouse model of acute MI, the injection of SPION-antiCD34/Nes caused a three-fold increase in CD133+ EPC accumulation at MI sites, as seen by MRI, compared to mice treated with phosphate-buffered saline. Furthermore, there was a notable improvement in the recovery of heart function. Using SPIONs to label stem cells made it possible to see their location and transplantation destiny, which improved treatment results [61].

To facilitate site-specific interactions and improve delivery to the target of interest, ligands can be conjugated to the surface of IONPs. Active targeting uses binding ligands attached to the material’s surface to specifically bind to the surface markers of a target cell or tissue, enhancing the specificity and efficiency of the contrast. Passive targeting depends on blood vessel leakiness and passive diffusion [60]. Targeting nanocarriers has advanced significantly recently to enhance therapeutic outcomes and reduce systemic toxicity.

Concluding this chapter, IONPs have enormous promise for various cardiovascular uses. Nanotechnology is currently only used in experimental, preclinical, or clinical research settings, but with continued effort and in-depth study, it is certain to raise the bar for CVD diagnosis and treatment.

## 3. Gold Nanoparticles

Thanks to their remarkable optical and surface plasmon resonance (SPR) qualities, gold NPs (AuNPs) are among the most widely used nanomaterials, especially in the biological and pharmaceutical industries. With their appealing physicochemical properties that serve as the foundation for significant light absorption and scattering, AuNPs are also attractive candidates for cardiovascular imaging [62]. The size and shape influence the function of nanoparticles. Specifically, particles smaller than 20 nm absorb light, whereas those larger than 80 nm cause strong light scattering. With the advantage of venous injection over arterial catheterization, gold provides 2.7 times more contrast than iodine, has a higher absorption, and has a longer circulation time [63]. These factors may help ensure appropriate circulatory system visualization by computed tomography (CT) scan [64]. As iodine cannot be functionalized, it is inappropriate for molecular imaging. Without ionizing radiation, photoacoustic imaging provides real-time detail acquisition with better tissue contrast than the US, higher spatial resolution, and deeper depth than fluorescence imaging [65]. Moreover, appealing properties like inert nature, non-toxicity, antibacterial activity, large surface area, and the possibility of functionalization recommend Au NPs as potential therapeutic platforms [66].

### 3.1. Therapeutic Platforms

The current medications used to treat heart diseases have reached their limits and have significant side effects when taken by patients for the rest of their lives to manage their symptoms and improve their prognosis [67]. It follows that optimizing the use of currently available medications and combining them with AuNPs to increase delivery efficiency is a sensible and straightforward decision. This conjugation is anticipated to provide current medications with “new and double-enhanced” potentiality as drug delivery into cardiac muscle cells becomes more precise and efficient in the conjugated form [68]. AuNPs’ large surface area-to-volume ratio makes it possible to coat hundreds of molecules, such as antifouling polymers, targeting agents, and therapeutics, on their surface. Since it is difficult to target the diseased area of the heart using traditional intravenous administration methods and it is dangerous to administer drugs directly into the heart, AuNPs carrying cell growth factors would help repair the damaged heart tissue resulting from coronary artery disease [23].

The classical structural model of a single AuNP is called a monolayer-protected cluster (MPC), and it is typically made up of an outer layer made of organic ligands or surfactants and a central inner gold atom known as the “core” [69]. The protective outer layer stabilizes gold nanoparticles and provides the surface for functionalization, while the inner core controls the structure’s crystallinity. AuNPs can form a variety of nanostructures, such as tetrahedrons, cubes, cylinders, icosahedrons, dodecahedrons, octahedrons, and many-branched structures, as well as clusters, catalytic particles, or plasmonic crystals [15,70]. Shevach et al. [71] recently deposited AuNPs on fibrous decellularized matrices to create a gold nanoparticle/scaffold. The cardiac cells grown inside gold–hybrid scaffolds showed elongated and aligned morphology and organized electrical coupling proteins (connexin 43), which may enhance the function of cardiomyocytes by stimulating the electrical signaling of cells.

Some studies added biologicals to the NP synthesis to decrease the risks to human health and the environment and improve biocompatibility. After the cyanobacterium incorporated the gold ions into the environment, Bakir et al. [72] synthesized AuNPs using *Lyngbya majuscula*. AuNPs, in conjunction with the cyanobacterium, have proven to be an effective management strategy for myocardial infarction in rat MI models. Certain phytochemicals in the cyanobacterial extract may cause synergistic activity; these phytochemicals may protect gold nanoparticles or change how the body processes and excretes them.

### 3.2. Computed Tomography Applications

While CT is an excellent tool for evaluating coronary lesions in cardiac imaging, it is unsuitable for measuring scar tissue’s transmural extent. Danila et al. created a novel strategy based on specific targeting of myocardial scar tissue to increase the detection sensitivity of scar tissue. Collagen targeting peptide (CNA35), a peptide against collagen-I, was used to coat AuNPs (40–70 nm) that were then injected into mice suffering from heart ischemia and allowed to recover for 30 days [73].

AuNPs may add value to a customized diagnosis if the conventional imaging modalities offer anatomical information. The use of AuNPs as a contrast agent in the cardiovascular field is still in its infancy despite promising preclinical results. Currently, no imaging modality provides the best results for clinical needs [62]. The myocardium of mice that had heart ischemia was shown to have extensive scar tissue with focal contrast enhancement in the CT and X-ray images. Histological staining provided additional validation for these findings [74]. This paper was the first to show how AuNPs coated with a collagen-homing peptide could be used in preclinical models to identify cardiac scar tissue using CT. This method could be used to identify atherosclerotic plaques and, consequently, assess the stability of the plaque since the fibrous cap in atherosclerotic plaque is primarily made of collagen and elastin [75].

A wide range of anatomical, functional, and molecular data could be obtained by developing multimodal imaging with AuNPs, which would help identify high-risk lesions and prevent future cardiovascular events. It was observed that multimodal imaging strategies that employ the same contrast agent allow for fewer contrast agent injections, improved targeted area contrast enhancement, and complementary disease information. In photoacoustic or optical coherence tomography (OCT) imaging, AuNPs can enhance the signal. However, a major drawback associated with light penetration into deep tissues is that although detecting atherosclerotic plaque in mice is relatively simple, in humans, it becomes more complex due to the deeper localization of the lesion (e.g., ischemic myocardium, clot, and atherosclerotic plaque). With an intravascular probe, this can be partially overcome [76]. Before their clinical implementation, more research and development of AuNPs as contrast agents are required. This entails a more thorough assessment of the preclinical model, the imaging modality, and the AuNPs (size, local concentration, targeted, or not). AuNPs should have a large imaging window and be able to be gradually excreted by the kidney for a potential cardiovascular clinical imaging translation (thereby avoiding repeated injection) [77]. Cheheltani et al. recently synthesized these kinds of AuNPs, which may find use in CT and photoacoustic imaging. Before translation into the clinic, evaluation of the contrast enhancement on large preclinical models (e.g., pigs) is crucial. In clinical settings, the location of the diseases, the resolution, and the sensitivity required for a better diagnosis and, consequently, an improved patient risk stratification should all be considered when selecting an imaging technique (invasive vs. non-invasive) using AuNPs as contrast agents [78].

To co-localize with active macrophages in plaques, gold nanoparticles have recently been used as contrast agents in combination with intravascular ultrasound and photoacoustic imaging [79]. The content, infiltration, and proliferation of macrophages could be identified using NP-based imaging. It gives clinical staff valuable information to track the development and brittleness of atherosclerotic plaques. Chhour et al. [80] produced AuNPs and labeled primary monocytes, which were injected into mice lacking apolipoprotein E. Their recruitment into atherosclerotic plaques was monitored using CT. An increase in attenuation was observed after these labeled cells were injected due to the recruitment of the AuNP-labeled monocytes into the plaques [80].

Cardiovascular imaging research is still breaking new ground, yet AuNPs may offer value beyond standard imaging modalities in terms of individualized diagnosis if they provide anatomical information [77].

## 4. Silver Nanoparticles

Silver nanoparticles (AgNPs) produce a pro-oxidant environment, though it is still unclear exactly how they increase reactive oxygen species (ROS). Some theories link the release and accumulation of intracellular Ag^+^ to the elevated production of ROS. Nevertheless, current data indicate that AgNPs themselves—rather than the released ions—lead to ROS production. Numerous growing pieces of evidence demonstrate the toxicity of AgNP on various body systems [81]. AgNPs have been shown to harm the skin, sperm cells, liver, bone marrow, lungs, spleen, kidneys, and vasculature. Evidence indicates that these NPs cause defective ubiquitination autophagosome–lysosome fusion at the subcellular level [82]. Future, in-depth studies on the genotoxicity of AgNPs and their potential role in cancer are now of interest, as evidenced by multiple studies [83]. Interestingly, although some research contends that these particles mainly spare the blood–brain barrier (BBB) from toxicity, other reports found that administering AgNP altered the BBB’s permeability, providing a fascinating potential avenue for future applications [15].

**Figure 3:**
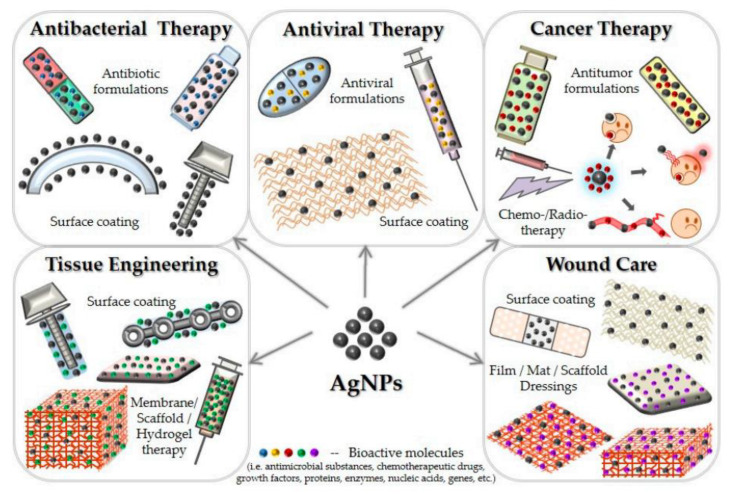
Different applications of AgNPs. Reprinted from an open-access source [85].

AgNPs have been widely used in food storage, environmental, biomedical, and household utensils, as well as in the healthcare sector, because of their unique qualities. Numerous book chapters and reviews have been written about the different applications of AgNPs, especially highlighting the numerous biological and biomedical uses of AgNPs, including their antibacterial, antifungal, antiviral, anti-inflammatory, anticancer, and anti-angiogenic properties [84,85,86,87]. Figure 3 provides a schematic diagram illustrating the different applications of AgNPs.

### 4.1. Antimicrobial and Anti-Inflammatory Effects

Numerous studies recently examined the impact of AgNPs on different cell types found in the intricate vascular system; however, the published outcomes were inconsistent [88]. Nonetheless, the gathered information can offer significant insights into the possible advantages of AgNPs for physiological and pathological phases associated with the cardiovascular system, thereby aiding in creating innovative and targeted molecular treatments in vascular tone, vasopermeability, and angiogenesis [89]. Cardiovascular diseases like hypertension may have an impact on the harmful effects of AgNPs. For instance, a prosthetic silicone heart valve coated with elemental silver was the first silver-modified cardiovascular medical device. It was created to prevent bacterial infections related to the valve and to lower the inflammatory response [90].

Since AgNPs have a well-established antibacterial effect, they are currently the most widely utilized nanomaterial in industrial and consumer goods. Additionally, silver has been used as an antimicrobial agent since ancient times in the ionic forms of silver chloride and silver nitrate, which are vital multifunctional medical products [91,92,93]. Even though the use of AgNPs has many benefits, there is significant concern about the effects these particles may have on human health because, upon release into the environment and contact with aqueous media (biological fluids), AgNPs oxidize from elemental Ag (0) to Ag^+^, and this may lead to Ag^+^ binding to ligands like Cl^−^ or cysteine and methionine from proteins. Since the amount of Ag^+^ released often determines the toxicity activity of AgNPs, all these reactions are highly relevant to the potential toxic effects of AgNPs [94].

The most prevalent cardiovascular condition that raises the global death rate is still MI. An important factor in the development of MI is the inflammatory response. Notably, it has been demonstrated that generating ROS can cause nuclear factor-kappa B (NF-kB)-mediated MI inflammation. A well-known model for examining the pathophysiology of myocardial ischemia is isoproterenol (Iso)-induced MI. Adrenergic in nature, Iso increases myocardial oxygen consumption by promoting heart rate and contractility. An overabundance of heart stimulation can cause ischemia, which can then result in MI. Myocytes experience anaerobic metabolism and reduced ATP synthesis during ischemia and infarction [95], compromising the membrane integrity and causing calcium overload and myocardial dysfunction. Following an ischemia insult, an abrupt oxygen supply frequently produces radical oxygen species, particularly by mitochondria, which further harms myocytes and causes necrosis. One transcription factor synthesized in the nucleus and transported into mitochondria is called mitochondrial transcription factor A (TFAM) [96]. It can reduce calcium mishandling in the myocardium brought on by Iso and functions by stabilizing mtDNA. Moreover, there is ample proof that oxidative stress and mitochondrial dysfunction accompany the decreased expression of PGC-1α and TFAM in numerous animal models of heart failure [97]. Targeting these genes for MI conditions will be appealing because NF-kB is also linked to the NOD-like receptor superfamily and the pyrin domain containing 3 (NLRP-3) inflammasome pathway, which are highly expressed in cardiac dysfunction.

### 4.2. Antioxidant Effects

Comparing the antioxidant and anti-inflammatory qualities of AgNPs to those of the traditional form of silver (i.e., AgNO_3_), Arozal et al. [98] examined the impact of AgNPs in the Iso-induced MI model in rats. Moreover, AgNPs will be examined between treatment groups, as they likely alter mitochondria biogenesis. The liver and kidney functions were examined as part of the AgNP safety profiles. The study also looked into the dysregulation of the mitochondria brought on by Iso. According to the findings, TFAM and PGC-1α mRNA expression levels were significantly raised after receiving 14 days of pretreatment with AgNPs rather than AgNO_3_, even though the expression levels of those mRNAs in the Iso group were similar to those in the normal group.

AgNO_3_ and AgNPs exhibit distinct effects, indicating that silver in the form of nanoparticles could only enter the nucleus or mitochondria and trigger the expression of TFAM and PGC-1α (peroxisome proliferator-activated receptor), which would then protect cardiac cells. Contrary to what many published articles claim, exposure to AgNPs can cause mitochondrial damage in cells, including swelling, disruption of mitochondrial membrane potentials, and apoptosis induced by the mitochondrial pathway [99]. In the case of AgNPs developed by Arozal et al. [98], the formulation showed advantageous effects attributed to the green synthesized method that uses alginate. By using lactate dehydrogenase (LDH) and creatine kinase-MB (CK-MB) activities, which are markers of cardiac enzyme leakage, they could show in this study that the administration of Iso in rats was linked to myocardial injury. When comparing the AgNO_3_ and AgNP groups to the Iso group, there was a significant decrease in CK-MB activity, suggesting that silver protects against myocardial damage. While both AgNO_3_ and AgNPs improved cardiac enzyme markers, only AgNPs could reverse the histological alterations in the myocardium brought on by Iso [98].

Through identifying cardiac markers, a recent study by Adibkia et al. [100] examines the in vitro impact of silver nanoparticles (Ag-NPs) on the cardiomyogenic differentiation of bone marrow-derived mesenchymal stem cells (BM-MSCs). To achieve this, MSCs were separated from bone marrow inhabitants and developed into cardiac cells in a special medium containing Ag-NPs. Additionally, immunocytochemistry was used to confirm that BM-MSCs had undergone cardiomyogenic differentiation. Then, the cells’ genes and proteins were evaluated, and the absolute telomere length (TL) was measured using real-time PCR and a Western blotting assay, respectively. It was discovered that in the cardiomyogenically differentiated BM-MSCs, 2.5 µg/mL Ag-NPs changed the expression of the genes and proteins VEGF, C-TnI, VWF, SMA, GATA-4, TERT, and cyclin D. They also caused elongation of the telomeres. Furthermore, as key players in pathways, there was a notable upregulation of Wnt3 and β-catenin’s protein and gene expression. Using the Wnt3/β-catenin signaling pathway, it was deduced that Ag-NPs could alter the in vitro expression of cardiac markers in BM-MSCs [100].

Conclusively, even if AgNPs show improved effects for CVD, further investigations are required regarding their toxicity and effect on the heart [101].

## 5. Cerium Oxide Nanoparticles

Another class of engineered nanomaterials studied for potential therapeutic applications comprises cerium dioxide nanoparticles (CeO_2_ NPs). CeO_2_ NPs have two valence states (Ce^3+^ and Ce^4+^), contributing to their catalytic activity. These nanoparticles’ oxygen vacancies allow them to react with the environment’s ROS, presenting CeO_2_ NPs as a possible in vivo mimetic for endogenous antioxidants like superoxide dismutase [102].

The irreversible oxidative states of CeO_2_ NPs—Ce^3+^ and Ce^4+^—which endow them with numerous special and physiologically significant characteristics, including the ability to scavenge free radicals and act as antioxidants, are drawing attention to them. Because of all these features, CeO_2_ is an excellent agent for treating various heart-related disorders [103,104,105,106]. Due to its multivalent oxidative state, cerium possesses anti-inflammatory, antioxidant, and radical-scavenging qualities, holding great promise for reducing oxidative stress [107,108,109]. CeNPs are also frequently utilized to treat infectious diseases, diabetes, and cancer [109,110], representing a valuable tool in neurology and immunology as well [111,112].

Oxidative stress is the primary cause of CVD, which in turn damages endothelial cells (ECs). Through endocytosis, these ECs can absorb nanoceria. Following entry, ECs will disperse throughout the cytoplasm and lower ROS levels, lowering the likelihood of apoptosis. Thus, cerium has a special quality that makes it possible to develop it as a novel treatment and diagnostic strategy for CVD [113].

Anomalies in the normal structure and function of the coronary microcirculation are indicated by microvascular dysfunction [114]. Cell dysfunction in smooth and endothelial muscles causes this. Coronary microcirculation controls coronary blood flow according to cardiac oxygen requirements. When this function is compromised, numerous cardiovascular anomalies arise [115]. Individuals with non-obstructive coronary artery disease (INOCA) and ischemia symptoms are more likely to experience coronary microvascular dysfunction [116]. Because CeO_2_ NPs significantly lower ROS species levels, they may be able to significantly lower the levels of microvascular dysfunction. Furthermore, CeO_2_ can significantly reduce endothelial-dependent vasodilation. In a study, 100 μg of CeO_2_ (≈0.42 mg/kg) was injected intravenously into rats that were spontaneously hypertensive (SH), which is a well-established model of microvascular dysfunction. Microvascular dysfunction showed a noteworthy improvement with CeO_2_ exposure coming in second (43.76 ± 4.33%) [115].

Although no evidence of protection in vivo has been found, CeO_2_ NPs have been demonstrated to shield cells in culture from deadly stress. Mice expressing monocyte chemoattractant protein (MCP)-1, specifically in their hearts, develop ischemic cardiomyopathy linked to the endoplasmic reticulum (ER) stress activation. According to research by Niu et al. [117], treatment with CeO_2_ NPs significantly reduced the levels of MCP-1, C-reactive protein, and total nitrated proteins in the serum and significantly inhibited the progressive dilatation and dysfunction of the left ventricle in MCP mice.

CeO_2_ NPs can also dramatically reduce the expression of pro-inflammatory cytokines, the buildup of 3-nitrotyrosine, the infiltration of monocytes and macrophages, and apoptotic cell death, including tumor necrosis factor (TNF)-α, interleukin (IL)-1β, and IL-6 in the myocardium. It was determined that CeO_2_ NPs inhibited the expression of important ER stress-associated genes, such as glucose-regulated protein 78 (Grp78), protein disulfide isomerase (PDI), and heat shock proteins (HSP25, HSP40, and HSP70) [118,119]. This suggests that CeO_2_ NPs may prevent the development of cardiac dysfunction and remodeling by reducing inflammatory processes, ER stress, and myocardial oxidative stress, which is most likely due to their auto-regenerative antioxidant qualities [117,120].

In a recent study, Guerra-Ojeda et al. [121] demonstrated that CeO_2_ NPs increase the expression of both SOD isoforms, which increases the bioavailability of NO through the reduction of $O_{2}^{-}$. Additionally, CeO_2_ NPs raise the expression of ACE2 in the human saphenous vein, which could explain why the contractile effect of angiotensin II is reduced when these nanoparticles are present. These findings may be helpful because endothelial dysfunction, linked to a drop in NO and a propensity for vasoconstriction, is a defining feature of cardiovascular disease. Conversely, a decrease in H_2_O_2_ production linked to bradykinin-induced vasodilation may result from the decrease in NOX4 expression brought on by CeO_2_NPs. This potent endothelium-dependent vasodilator, bradykinin, is also a contractile agonist in nonvascular smooth muscle and is related to edema, inflammation, and the pain mechanism, despite the initial impression that the nanoparticles have a negative effect by reducing the vascular response to bradykinin. Thus, in this context, CeO_2_NPs reduced the response to bradykinin, which may be advantageous for pain or inflammation relief [121].

Research on CeO_2_ NP has yielded inconsistent findings regarding the effectiveness of its possible antioxidant activity, which is similar to that of many other pharmaceuticals. Following the co-incubation of CeO_2_ NPs, in vitro analysis has demonstrated increased ROS generation and inflammation; however, other studies using cardiac progenitor and endothelial cells have decreased both parameters. Even more intricate is the in vivo examination of these nanoparticles. It has been experimentally demonstrated that injecting CeO_2_ NPs can reduce tissue damage, which is frequently linked to radiation therapy and strokes. Even with these encouraging outcomes, injecting CeO_2_ NPs into young, healthy rats may have negative consequences that lead to microvascular dysfunction [122]. It is unclear at this time whether CeO_2_ NPs’ possible benefits are pathology-specific.

Moreover, the impact of CeO_2_ NP-specific changes in ROS production on microvascular function remains unclear. According to Minarchick et al. [102], injectable CeO_2_ NPs would lessen oxidative stress and microvascular dysfunction linked to hypertension. To mimic a therapeutic application, intravenously administered saline or CeO_2_ NP (100 μg suspended in saline) was given to spontaneously hypertensive (SH) and Wistar–Kyoto (WKY) rats. Intravital microscopy measured the mesenteric arteriolar reactivity 24 h after exposure. The dependent and independent endothelium functions were evaluated using sodium nitroprusside and acetylcholine, while fluorescence staining was used to examine microvascular oxidative stress in isolated mesenteric arterioles. In a high ROS environment, this study shows that injected CeO_2_ NP reduces microvascular oxidative stress, which enhances microvascular function. These findings add to the knowledge of CeO_2_ NPs behavior in vivo and demonstrate their antioxidant therapeutic potential, especially in conditions with increased reactive oxygen species. This study also demonstrated alterations in the inflammatory profile after exposure to CeO_2_ NPs. Similarly, it makes sense to hypothesize that the proactive use of CeO_2_ NPs would prevent the abrupt rise in local ROS levels linked to different injuries and medical interventions. The WKY group was the primary recipient of these alterations, emphasizing the necessity of additional study on these nanoparticles to completely comprehend their effects on systemic inflammation [102].

A potentially helpful autologous cell source for cardiac regenerative medicine is cardiac progenitor cells (CPCs). Reactive oxygen species must be maintained at physiological levels in vitro. However, CPCs culture in vitro requires the presence of microenvironmental conditions (a complex array of bioactive substance concentration, mechanostructural factors, and physicochemical factors) that closely mimic the natural cell surrounding in vivo [123]. Because they are redox-active, CeO_2_ NPs, or nanoceria, may be a powerful tool for reducing oxidative stress in isolated CPCs [124]. In a recent publication, Pagliari et al. demonstrated that 24 h of exposure to 5, 10, and 50 μg/mL of nanoceria did not impact cardiac progenitor cell growth and function [125]. However, it was able to shield CPCs from H_2_O_2_-induced cytotoxicity for a minimum of 7 days, suggesting that nanoceria is an efficient antioxidant. Compared to related controls and in the context of the current investigation, the internalized nanoceria particles—inert regarding CPC homeostasis and differentiation—remain ostensibly silent inside CPCs and eventually serve as a defense against oxidative insults [126]. Redox cycles between the Ce^3+^ and Ce^4+^ oxide states that react with superoxide and hydrogen peroxide, imitating the actions of the two main antioxidant enzymes, catalase and SOD, may be the cause of the reduction in intracellular ROS in CeO_2_ NPs [127].

Therefore, understanding how CeO_2_ NPs act in vivo in low and high-ROS environments is critical for this potential therapeutic agent’s continued and expanded development [102].

## 6. Toxicity of Metal-Based Nanoparticles

The widespread manufacturing and use of nanomaterials has led to the ongoing release of nano-sized particles into the environment, and it is impossible to overlook the health hazards that exposure to these materials poses to the general public and those in the workforce. Research has indicated a strong correlation between cardiovascular disease and particle exposure [128]. Experiments conducted in vivo and in vitro have shown that nanoparticles can cross the alveolar–capillary barrier and enter the bloodstream and systemic organs. The information presented above suggests that the heart is a specific target organ where nanoparticles can build up and harm the heart [129].

Regarding iron oxide nanoparticles, it was recently discovered that when iron oxide nanoparticles-tagged stem cells were injected locally intramyocardially into rats, iron may accumulate in the infarcted myocardium for at least six months. Thus, it makes sense to assume that the local myocardial administration of IONPs-mediated therapeutic or diagnostic drugs could result in myocardial iron overload, exacerbating the ischemia myocardium’s unfavorable remodeling and worsening the heart’s ability to function [130]. Low dosages of these NPs are less harmful, but acute exposure causes phenotypic changes in ECs, oxidative stress, apoptosis, endothelial activation, endothelial dysfunction, and NOS dysfunction because of their tiny particle size and vast surface area. Heart failure, coronary heart disease, and atherosclerotic hypertension are intimately associated with these impairments [131]. Fe_3_O_4_ NPs with diameters of 2.3, 4.2, and 9.3 nm were created by Wu et al., and their toxicity was assessed in mice through intravenous injection. The findings show that at a dosage of 100 mg/kg, ultrasmall iron oxide nanoparticles with small sizes (2.3 and 4.2 nm) were extremely hazardous. In contrast, iron oxide nanoparticles measuring 9.3 nm in size exhibited no overt toxicity. When the entire exposure was divided into 4 doses, each lasting 5 min, the toxicity of tiny nanoparticles (2.3 and 4.2 nm) might be decreased [54]. The detrimental effects of prolonged exposure to Fe_2_O_3_ NPs and AgNPs on the heart and lungs were determined in a study reported by Yousef et al. Different routes, such as oxidative DNA alteration, production of inflammation, creation of free radicals, and suppression of antioxidant systems, were used to cause lung damage and cardiotoxicity. Moreover, NPs change the lipid profiles and histology of the heart and lungs. Moreover, the outcomes clearly showed that co-exposure to Fe_2_O_3_NPs and AgNPs simultaneously had more detrimental effects on the heart and lungs than individual NP exposure [91].

In reference to gold nanoparticles, Zhang et al. administered various sized Au NPs to rats with hyperthyroid heart disease (HHD) that was induced by isoproterenol (ISO). Intravenous injection of 5, 40, and 100 nm AuNPs was performed as a single dose. Heart marker enzymes in serum were used to assess cardiac safety assessments, and ICP-MS was used to determine the buildup of AuNPs in the heart. The findings demonstrated the size-dependent cardiac effects of AuNPs produced by ISO in rats with hyperthyroidism. Because 5 nm AuNPs are smaller than larger AuNPs and can more readily adapt to the entire body in vivo, they may have some cardiac protective effects but less accumulation in the heart. AuNPs can cause pathological changes in control groups, such as cardiac fibrillation and apoptosis, according to histological examination and TUNEL staining; however, they can shield HHD groups from these negative effects [132,133]. Moreover, Abdelhaim conducted a study that found that when compared to the corresponding control rats, the AuNPs-treated rats received 100 μL of 10 and 20 nm particles for 3 or 7 days, showing congested heart muscle with prominent dilated blood vessels, scattered and extravasations of red blood cells, focus of muscle hyalinosis, disturbed muscle fascicles, a dense prominent focus of inflammatory cells infiltrated by small lymphocytes and few plasma cells. On the other hand, the AuNPs-treated rats received 100 μL of 50 nm particles for 3 or 7 days, showing benign-looking heart muscle with normal muscle direction and fascicles and very few scattered small lymphocytes. This suggests that the histological alterations caused by the intraperitoneal administration of AuNPs were size-dependent and related to time exposure of AuNPs [134,135]. In a different study, it was discovered that growing zebrafish embryos exposed to 50 and 100 mM of Au 1.4MS could develop a string-like heart in addition to other abnormalities. The 1.4 nm AuNPs failed electrophysiology-based safety assessments in a patch clamp experiment with human embryonic kidney cell line 293 cells that expressed the human ether-a-go-go-Related gene (hERG). Even though hERG channels can be blocked by ultrasmall AuNPs in vitro, a large dose of 50 mg/kg in vivo did not block the channel or cause even a slight arrhythmia in the heart [23,136].

It has been shown that Ag ionic form, in concentrations of 1 part per thousand in drinking water (57 and 68 mg of silver/kg of body weight/day), induces cardiac alterations in rats, such as left ventricular hypertrophy, in the context of the cardiovascular system [137]. Additionally, it has been demonstrated that feeding turkeys 900 ppm of silver nitrate for four weeks (110 mg/kg of body weight per day) increased the birds’ hematocrit, hemoglobin concentrations, aortic elastin content, and heart size. The authors of these studies proposed that silver deposits in the basement membranes of renal glomeruli and elevated blood pressure were the causes of heart hypertrophy. Since female rats exposed to AgNPs (60 nm; 300 mg/kg of bw/day) also experienced an increase in hematocrit and hemoglobin concentrations following a 28-day oral exposure, the effects of Ag^+^ are equivalent to those of AgNP [13]. Moreover, a study by Lin et al. examined the possible toxicity of AgNPs, which is rarely studied, on cardiac electrophysiology. In vitro studies on mouse cardiac papillary muscle cells revealed that AgNPs (10^−9^–10^−6^ g/mL) concentration-dependently depolarized the resting potential, reduced the action potential, and ultimately resulted in a loss of excitability. AgNPs (10^−9^–10^−7^ g/mL) concentration-dependently reduced the Na^+^ currents (I_Na_), sped up the activation, postponed the inactivation, and allowed the Na^+^ channels to recover from inactivation in 5 min in cultured newborn mouse cardiomyocytes. In addition, AgNPs at 10^−8^ g/mL quickly reduced the inwardly rectifying K^+^ currents (I_K1_) and postponed the I_K1_ channels’ activation. AgNPs injected intravenously at a dose of 3 mg/kg simply lowered heart rate; at a dose of ≥4 mg/kg, they produced cardiac asystole, full atrioventricular block, and sinus bradycardia [138,139]. Using isolated perfused hearts from spontaneously hypertensive rats (SHRs), Ramirez-Lee et al. sought to assess perfusion pressure (PP) and left ventricular pressure (LVP) as physiological markers of cardiovascular function in response to AgNPs and to determine the roles of NO and oxidative stress. The findings imply that 15 nm AgNPs enhanced oxidative stress and decreased the NO produced by endothelial/inducible NO-synthase, which enhanced and prolonged vasoconstriction and myocardial contractility. Furthermore, the traditional actions of acetylcholine (ACh) and phenylephrine (Phe) are modified by the hypertension situation. These findings imply that AgNPs-induced cardiac damage was amplified by hypertension [140,141]. Additionally, Espinosa-Cristobal et al. [142] noted higher concentrations of hematocrit and hemoglobin in female rats that were given drinking water containing AgNPs (14 and 36 nm) at a dose of 535 mg/l for 25 days (equivalent to 65 mg/kg of body weight per day). Since the results are similar, these data suggest that AgNP effects might involve the release of Ag^+^. Nevertheless, more research methods are needed, as there are not many studies examining the direct effects of AgNPs and Ag^+^ in cardiac physiology, which would enable us to establish and comprehend the mechanism of these NPs’ potential toxicity [142].

## 7. Conclusions and Future Perspectives

To summarize, the biological application of nanotechnology is becoming critical for treating CVDs, significantly impacting medicine in terms of monitoring, diagnosing, preventing, healing, or repairing diseases and injured tissues in biological systems. Nonetheless, raising awareness and advancing the use of nanotechnology is still crucial.

Metal-based nanoparticles are widely used in engineering and the biological sciences. This review gave a broad overview of the biological and physicochemical characteristics of the most commonly employed metal-based NPs and their use in CVDs. These particles offer great potential for CVD imaging and therapy because of their distinct size, physical characteristics, and chemical makeup. Still, specific worries could prevent them from being widely used in biomedicine. An extensive, expensive, and complicated process known as clinical translation and an industrial technology transfer phase are required before the medical nanomaterials can be administered to patients. The true benefits of the new technology must be weighed against the cost of production.

Furthermore, human efficacy in disease models is often inferior to animal models due to the significant physiologic differences between humans and small animals. The most crucial thing to remember is that metal-based NPs’ biological behavior and toxicological characteristics must be carefully evaluated. To evaluate their toxicity in vitro and in vivo, efforts must be made to standardize the methods. It is essential to create tested models that can forecast the release, transport, transformation, accumulation, and uptake of these nanostructures in humans and calculate their effects on vulnerable populations.

Moreover, improvements in CVD management should be sought by implementing and optimizing certain patents related to nanotechnological approaches. For example, cerium oxide nanoparticles have been patented for treating and preventing stroke and cardiovascular disease [143]. Therefore, this benchmark could serve as a promising inception point for additional advancements in the field.

## Figures and Tables

**Table 1:** Types of iron oxide nanoparticles and their applications.

Iron Oxide Nanoparticles	Applications
Fe_3_O_4_-PFH-DiR@CS-DS NPs	Magnetic resonance imaging, near-infrared fluorescence (NIRF)
2,3-dimercaptosuccinic acid modified Fe_2_O_3_ NPs	Protection of the heart from ischemic damage
IONPs surface modified with dextran	Macrophages infiltration
Magneto fluorescent nanoparticles (cross-linked iron oxide [CLIO]-Cy5.5)	MRI and fluorescence tomography
Fe@CNPs	Penetration of the internal structures of atherosclerotic plaques
Ultrasmall superparamagnetic particles of iron oxide	Improvement of the MRI
SPION-antiCD34/Nes	Increase CD133+ EPC accumulation at MI sites

## Data Availability

Not applicable.
